# New spatial records of vascular plants in the Azores Archipelago: the PRIBES project and the Azorean Biodiversity Portal (ABP) initiatives - II. Pico Island

**DOI:** 10.3897/BDJ.14.e206276

**Published:** 2026-08-10

**Authors:** Andrea Petrone, Paulo A. V. Borges, Fernando Pereira, Rui B. Elias

**Affiliations:** 1 University of Azores, CE3C—Centre for Ecology, Evolution and Environmental Changes, Azorean Biodiversity Group, CHANGE —Global Change and Sustainability Institute, School of Agricultural and Environmental Sciences, Rua Capitão João d’Ávila, Pico da Urze, 9700-042, Angra do Heroísmo, Azores, Portugal University of Azores, CE3C—Centre for Ecology, Evolution and Environmental Changes, Azorean Biodiversity Group, CHANGE —Global Change and Sustainability Institute, School of Agricultural and Environmental Sciences, Rua Capitão João d’Ávila, Pico da Urze, 9700-042 Angra do Heroísmo, Azores Portugal; 2 IUCN SSC Monitoring Specialist Group, Angra do Heroísmo, Azores, Portugal IUCN SSC Monitoring Specialist Group Angra do Heroísmo, Azores Portugal

**Keywords:** Azores Archipelago, ecology, endemism, floristic inventory, habitat diversity, introduced species, invasive species, island biogeography, monitoring, native species, Pico Island, vascular plants

## Abstract

**Background:**

Although the Azores Archipelago is widely recognised for its remarkable natural heritage, it is increasingly threatened by the rapid spread of invasive species, which are causing significant biodiversity decline and impairing ecosystem functioning and services. These biological invasions — mainly driven by intensified global trade and human mobility — also have consequences for public health, local livelihoods and economic activities.

To address these challenges, the PRIBES project ("Estratégia regional para o controlo e prevenção de espécies exóticas invasoras"; LIFE IP AZORES NATURA - LIFE17 IPE/PT/000010) advocates the implementation of an integrated regional strategy for the management of terrestrial and freshwater invasive species. The proposed framework focuses on coordinated governance through a dedicated task force, strengthened preventative actions including biosecurity measures and risk analyses and early detection systems supported by monitoring programmes and citizen-science initiatives. It also promotes a hierarchical management approach, ranging from eradication to long-term control and mitigation, according to feasibility and economic sustainability.

This strategy is supported by scientific research aimed at understanding invasion pathways and evaluating management outcomes, as well as collaborations at national and international levels that encourage data exchange and shared expertise. Public engagement is fostered through awareness campaigns and educational activities. In parallel, the Azores Bioportal functions as a key open-access repository for biodiversity information, providing validated species records and connections with international biodiversity networks. Together, these initiatives contribute to monitoring both native and invasive species, while offering valuable resources for conservation planning and evidence-based decision-making.

In the scope of the PRIBES Project and the Azores Bioportal, we started a series of data papers with new spatial records of vascular plants gathered during field campaigns across several Azorean Islands between 2019 and 2025. The first focused on São Jorge Island, whereas the second, current data paper focuses on Pico Island.

**New information:**

On Pico Island, a total of 251 vascular plant taxa were recorded, encompassing six classes, 38 orders and 88 families. These records correspond to 11,023 individual plant occurrences that have been recorded, including repeated observations of the same species across different sites. As each photographic observation is associated with unique geographic coordinates, all recorded specimens represent new spatial records for the island’s flora. Amongst the recorded taxa, 59 are endemic to the Azores, 68 are native, 123 are introduced and one is of uncertain status (*Dracaena draco* (L.) L.), corresponding to 6,019 endemic, 3,378 native, 1,624 introduced and two indeterminate occurrences. At the family level, 34 families include endemic taxa, 36 include native taxa, 60 include introduced taxa and one includes taxa of uncertain origin; because several families contain taxa belonging to different floristic status categories, these counts are not mutually exclusive. The inventory includes several Azorean endemic taxa, noteworthy records comprise *Sanicula azorica* Guthnick ex Seub., *Bellis azorica* Hochst. ex Seub., *Leontodon filii* (Hochst. ex Seub.) Paiva & Ormonde, *Scabiosa nitens* Roem. & Schult., Carex pilulifera subsp. azorica (J. Gay) Franco & Rocha Afonso, *Platanthera micrantha* (Hochst. ex Seub.) Schltr., *Dryopteris crispifolia* Rasbach, Reichst. & Vida and *Isoëtes azorica* Durieu ex Milde.

## Introduction

**Figure 1.**
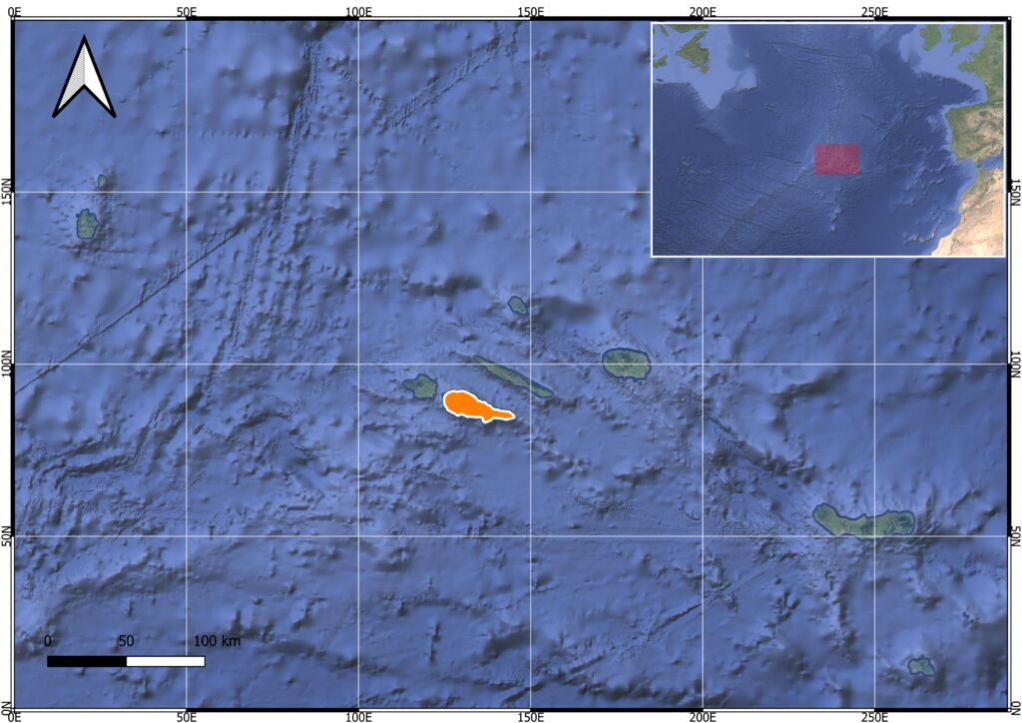
The Azores in the Atlantic Ocean, with Pico highlighted in orange (credit: Andrea Petrone).

The Azores Archipelago lies in the North Atlantic Ocean (ca. 37–40° N, 25–31° W), approximately 1,600 km west of mainland Portugal and forms part of the Macaronesian biogeographic region (Fig. 1). This Archipelago is composed of nine volcanic islands distributed into three groups: Western, Central and Eastern. These islands originated from volcanic activity associated with the divergence of the Eurasian, African and North American tectonic plates along the Mid-Atlantic Ridge (Ferreira 2005, Azevedo and Portugal Ferreira 2006, Fernández-Palacios et al. 2011, Connor et al. 2012).

**Figure 2.**
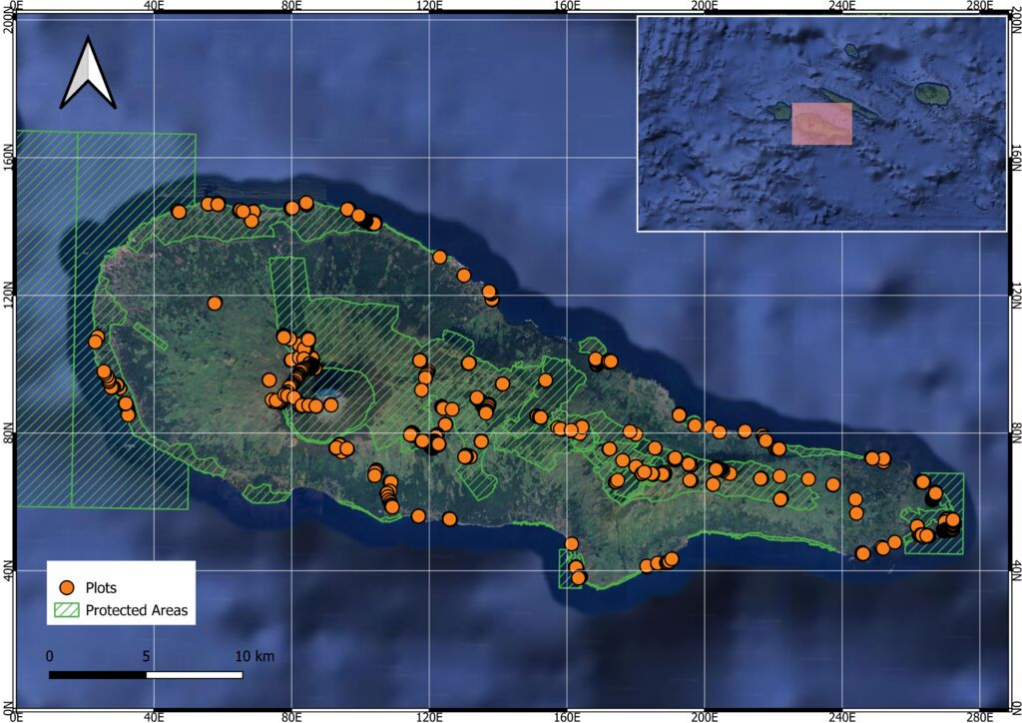
Georeferenced photographic plots (orange points) distributed across Pico Island (Azores). Protected areas are shown in green hatching (data source: UNEP-WCMC IUCN 2026, www.protectedplanet.net) (credit: Andrea Petrone).

The present study was conducted on Pico Island (Fig. 2), one of the islands of the Central Group. Pico Island is the second largest island of the Archipelago (ca. 447 km²), with an elongated E–W shape (ca. 42 km long and 15 km wide) and hosts the highest elevation in Portugal, reaching 2351 m a.s.l. at Mount Pico (Cancela d'Abreu et al. 2005, Fernandes et al. 2014). It is also the youngest island of the Archipelago (ca. 0.27 Ma), having developed within the Azorean microplate between the Eurasian and African plates (Zbyszewski 1963, Dias 1996, Azevedo and Portugal Ferreira 2006, Connor et al. 2012). This work represents the second paper of the project, following the publication of new spatial records for São Jorge Island.

The geomorphology of Pico Island strongly reflects its volcanic origin and tectonic structure. Three main volcanic units can be distinguished: (i) the older Topo volcano, a central shield volcano composed of basaltic and ankaramitic lava flows and partially dismantled by landslides and faulting; (ii) an intermediate volcanostratigraphic unit consisting of basaltic spatter cones aligned along WNW–ESE tectonic structures; and (iii) the youngest Madalena Volcanic Complex, including fissural systems and the Pico stratovolcano, which features a summit pit crater (Madeira 1998, Nunes 1999, Madeira and Brum da Silveira 2003, França et al. 2006, Dias et al. 2007, Fernandes et al. 2014). Historical volcanic activity (1562–64, 1718 and 1720) produced recent lava fields locally known as “Misterios”. Due to its volcanic origin, Pico is characterised by rugged topography shaped by lava flows, pyroclastic deposits and volcanic structures. Soils are generally young and poorly-developed andisols, while organic soils and peat deposits occur in topographic depressions and high-altitude environments (Dias 1996). Agricultural suitability is extremely limited, with only about 2.2% of the island considered arable without major constraints. Most soils exhibit severe limitations, being suitable mainly for natural pastures, forest or conservation purposes. This contributes to the relatively low intensity of land use, the sparse population and the persistence of large areas of natural and semi-natural habitats (Fernandes et al. 2014, Fonseca et al. 2014, Moreira et al. 2018).

From a climatic perspective, the Azores are characterised by a temperate oceanic climate, with high humidity, persistent cloud cover and mild temperatures throughout the year, with an average of approximately 17.5°C at sea level (Schaefer 2003). The climate of Pico reflects these general conditions, but is strongly modulated by altitude. Mean annual temperature is around 17.4–17.5°C at sea level, with limited seasonal variation. Precipitation is abundant and increases markedly with elevation, ranging from approximately 1000–1900 mm at low altitudes to over 4000 mm above 700 m. Rainfall is distributed throughout the year, with maxima in winter (January–February) and minima in summer (July). Horizontal precipitation from fog and cloud interception plays a crucial role, particularly between 180 m and 700 m, where persistent cloud cover is common. Relative humidity averages around 80% and increases with altitude, while snowfall may occur above 2000 m. Prevailing winds are predominantly from the southwest (Azevedo 1998, Azevedo et al. 1999).

As a mountainous oceanic island, Pico exhibits strong altitudinal gradients that shape its vegetation patterns. Although the Azores originally supported extensive native woody vegetation, human colonisation over recent centuries has substantially transformed the landscape through forest clearance for agriculture and pasture, the extraction of wood for fuel and construction and the introduction and spread of alien species, which have led to habitat loss, species replacement and biological invasions (Martins 1993, Silva and Tavares 1997, del Carmen Machado Yanes et al. 1997, De Nascimento et al. 2009, Triantis et al. 2010, Fernández-Palacios et al. 2011, Connor et al. 2012, Fernandes et al. 2014).

Despite these transformations, Pico retains some of the best-preserved natural habitats in the Azores, partly due to limited agricultural potential and low urbanisation, largely confined to coastal settlements such as Madalena, Lajes do Pico and São Roque. The Azores exhibit a well-defined zonal vegetation pattern, shaped by climatic gradients along elevation, from coastal communities to alpine vegetation. In Pico, this gradient is particularly well developed and includes: *Erica–Morella* coastal woodlands (0–100 m), *Picconia–Morella* lowland forests (ca. 100–300 m), *Laurus* submontane forests (300–600 m) (also known as Laurisilva in the Macaronesian Islands), *Juniperus–Ilex* montane forests (600–900 m), *Juniperus* montane woodlands (700–1000 m), *Calluna–Juniperus* altimontane scrublands (900–1100 m), *Calluna–Erica* subalpine scrublands (1200–1700 m) and *Calluna* alpine scrublands above 1700 m (Elias et al. 2016). The zonal vegetation of Pico follows a well-defined altitudinal sequence driven by climatic gradients, ranging from coastal *Erica–Morella* woodlands to high-elevation alpine scrublands. Pico is unique within the Azores in preserving nearly the complete sequence of vegetation belts, resulting in high ecological heterogeneity and the highest plant diversity of the Archipelago. High-altitude ecosystems remain relatively undisturbed, whereas lowland forests are fragmented and montane areas are partially affected by land-use change (Elias et al. 2016).

Comprehensive plant inventories and georeferenced occurrence datasets are essential for understanding island biogeography, monitoring ecological changes and supporting conservation strategies in the Azores (Whittaker et al. 2005, Borges et al. 2016, Elias et al. 2024, Petrone et al. 2026a, Petrone et al. 2026b).

This work contributes to the documentation and monitoring of the Azorean vascular plants within the PRIBES project and the Azorean Biodiversity Portal (ABP) initiative (Borges et al. 2010). The ABP is a regional biodiversity database compiling georeferenced records of marine and terrestrial taxa from historical literature, collections and field observations. The dataset presented here improves the spatial knowledge of plant distributions on Pico Island, supporting long-term monitoring and biogeographical studies.

## General description

### Purpose

Within the scope of the PRIBES project (see also Borges et al. (2022)), the aim of this study was to develop a spatially-explicit inventory of exotic potentially invasive plant species across diverse natural and semi-natural areas of Pico island (Azores).

### Additional information

This work was also part of a collaboration with the PORBIOTA projet, aiming to map the distribution of Azorean biodiversity in the AZORES BIOPORTAL.

## Project description

### Title

Mapping Vascular Plant Diversity on Pico Island under the PRIBES project.

### Personnel

The original project was conceived by Rui B. Elias and Paulo A.V. Borges.

Fieldwork (site selection and experimental setting): Rui B. Elias.

Fieldwork (authorisation): Azorean Regional Directorate for the Environment.

Fieldwork: Fernando Pereira and Rui B. Elias.

Taxonomists: Andrea Petrone and Rui B. Elias.

Darwin Core Databases: Andrea Petrone and Paulo A.V. Borges.

### Funding

Direcção Regional do Ambiente - PRIBES (LIFE17 IPE/PT/000010) (2019-2020). PORBIOTA - “ACORES-01-0145-FEDER-000072 - AZORES BIOPORTAL”, funded by the Operational Programme Azores 2020 (85% ERDF and 15% regional funds) (2019-2021). RBE and PAVB are currently funded by FCT through national and European funds by UID/00329/2023 - Centre for Ecology, Evolution and Environmental Changes (CE3C) and this project was also funded by M1.1.A/FUNC.UI&D/021/2025 [UI&D/GBA/2025], was funded by the Regional Directorate for Science, Innovation and Development [Regional Government of the Azores] through the PROSCIENTIA Incentive System (M1.1.A/FUNC.UI&D/021/2025 [UI&D/GBA/2025]).

## Sampling methods

### Study extent

This study was conducted across different areas of the volcanic island Pico (Azores), ranging from coastal areas to high-elevation humid forests, scrublands and grasslands, capturing a variety of habitat types, vegetation density and plant abundance.

### Sampling description

Fieldwork was conducted across multiple periods: August 2020, July 2021, June–July 2022 and June–July 2024. Georeferenced photographs were collected using a Garmin Montana 750i GPS unit, with Multi-GNSS (Global Navigation Satellite System) support and WAAS/EGNOS correction systems that enhance GPS signals with improved accuracy (2-3 m), integrity (error checking) and availability in open-sky conditions, documenting plant diversity across an altitudinal and ecological gradient — from coastal vegetation to high-elevation humid forests, scrublands and grasslands. Survey areas were selected to avoid highly disturbed habitats, such as urban zones, intensive pastures and exotic tree plantations. Photographs were taken mainly along trails, slopes, cliffs and road margins. Areas with a high degree of anthropogenic disturbance (e.g. intensive pastures, *Cryptomeria* plantations or urban areas) were avoided. Species visible in the images were identified post-fieldwork by expert taxonomists using floristic guides (e.g. Schaefer (2021), Elias et al. (2024)) and online resources (e.g. ABP (2026), Flora-On (2026), POWO (2026)).

Using georeferenced photographs in vegetation surveys provides crucial spatial and ecological information, especially if one needs to survey large areas over a limited amount of time. This approach provides precise geo-locations, enables long-term monitoring and ground truthing (verifying the accuracy of data collected remotely) and is also very important for nature conservation purposes. It can be used for tracking invasive species spread, monitoring post-fire regeneration, document phenology (flowering, fruiting stages) across seasons or recording rare or threatened plant populations with precise location data.

### Quality control

Species taxonomic rank and conservation status were assigned, based on: a) the information available through the Azores Bioportal – PORBIOTA (https://azoresbioportal.uac.pt/pt/) (see also Elias et al. (2024)); b) the official biodiversity platform for the Azores Archipelago, which integrates validated and up-to-date data on the region’s flora (including endemism, invasiveness and habitat preference).

## Geographic coverage

### Description

Pico in the Azores (Figs 1, 2)

### Coordinates

38°23'14.6''N and 38°33'37.55''N Latitude; 28°32'23.28''W and 28°1'44.87''W Longitude.

## Taxonomic coverage

### Description

Kingdom: Plantae

Phylum: Tracheophyta

Classes: Liliopsida, Lycopodiopsida, Magnoliopsida, Pinopsida, Polypodiopsida, Selaginellopsida.

Orders: Alismatales, Apiales, Aquifoliales, Asparagales, Asterales, Boraginales, Brassicales, Caryophyllales, Commelinales, Cornales, Cyatheales, Dipsacales, Ericales, Fabales, Fagales, Gentianales, Hymenophyllales, Isoëtales, Lamiales, Laurales, Liliales, Lycopodiales, Malpighiales, Malvales, Myrtales, Osmundales, Pinales, Poales, Polypodiales, Ranunculales, Rosales, Santalales, Sapindales, Saxifragales, Selaginellales, Solanales, Vitales, Zingiberales.

## Usage licence

### Usage licence

Creative Commons Public Domain Waiver (CC-Zero)

## Data resources

### Data package title

Mapping Vascular Plant Diversity on Pico Island under the PRIBES project

### Resource link

https://doi.org/10.15468/vk4y7k

### Alternative identifiers

https://www.gbif.org/dataset/f40f80dc-1815-4e73-9097-9d7893d87f6f#

### Number of data sets

2

### Data set 1.

#### Data set name

Event Table

#### Data format

Darwin Core Archive format

#### Character set

UTF-8

#### Download URL

https://ipt.gbif.pt/ipt/resource?r=pico_vascular_plants

#### Data format version

1.10

#### Description

The dataset was published in the Global Biodiversity Information Facility platform, GBIF (Petrone et al. 2026b). The following data-table includes all the records for which a taxonomic identification on species level was possible. The dataset submitted to GBIF is structured as a sample event dataset that has been published as a Darwin Core Archive (DwCA), which is a standardised format for sharing biodiversity data as a set of one or more data tables. The core data file contains 2801 records for Pico island (eventID). This GBIF IPT (Integrated Publishing Toolkit, Version 2.5.6) archives the data and, thus, serves as the data repository. The data and resource metadata are available for download in the Portuguese GBIF Portal IPT (Petrone et al. 2026b).

**Data set 1.** 

Column label	Column description
eventID	Identifier of the event, unique for the dataset.
datasetName	The name identifying the dataset from which the record was derived.
continent	The name of the continent in which the dcterms:Location occurs.
stateProvince	The name of the next smaller administrative region than country (state, province, canton, department, region etc.) in which the dcterms:Location occurs.
islandGroup	The name of the island group in which the dcterms:Location occurs.
island	The name of the island on or near which the dcterms:Location occurs.
country	The name of the country or major administrative unit in which the dcterms:Location occurs.
countryCode	The standard code for the country in which the dcterms:Location occurs.
municipality	The full, unabbreviated name of the next smaller administrative region than county (city, municipality etc.) in which the dcterms:Location occurs. Do not use this term for a nearby named place that does not contain the actual dcterms:Location.
minimumElevationInMetres	The lower limit of the range of elevation (altitude, usually above sea level), in metres.
decimalLongitude	The geographic longitude (in decimal degrees, using the spatial reference system given in dwc:geodeticDatum) of the geographic centre of a dcterms:Location. Positive values are east of the Greenwich Meridian, negative values are west of it. Legal values lie between -180 and 180, inclusive.
decimalLatitude	The geographic latitude (in decimal degrees, using the spatial reference system given in dwc:geodeticDatum) of the geographic centre of a dcterms:Location. Positive values are north of the Equator, negative values are south of it. Legal values lie between -90 and 90, inclusive.
habitat	Description of the habitat where the specimen was found.
geodeticDatum	The spatial reference system upon which the geographic coordinates are based.
coordinateUncertaintyInMetres	Uncertainty of the coordinates of the centre of the 50 m transect in metres.
coordinatePrecision	A decimal representation of the precision of the coordinates given in the dwc:decimalLatitude and dwc:decimalLongitude.
georeferenceSources	A list (concatenated and separated) of maps, gazetteers or other resources used to georeference the Location, described specifically enough to allow anyone in the future to use the same resources.
day	The integer day of the month on which the dwc:Event occurred.
month	The integer month in which the dwc:Event occurred.
year	The four-digit year in which the dwc:Event occurred, according to the Common Era Calendar.
eventDate	The date-time or interval during which a dwc:Event occurred. For occurrences, this is the date-time when the dwc:Event was recorded. Not suitable for a time in a geological context.
sampleSizeValue	A numeric value for a measurement of the size (time duration, length, area or volume) of a sample in a sampling dwc:Event.
sampleSizeUnit	The unit of measurement of the size (time duration, length, area or volume) of a sample in a sampling dwc:Event.
eventRemarks	Comments or notes about the dwc:Event.
samplingProtocol	The names of, references to or descriptions of the methods or protocols used during a dwc:Event.

### Data set 2.

#### Data set name

Occurrence Table

#### Data format

Darwin Core Archive format

#### Character set

UTF-8

#### Download URL

https://ipt.gbif.pt/ipt/resource?r=pico_vascular_plants

#### Data format version

1.10

#### Description

The dataset was published in the Global Biodiversity Information Facility platform, GBIF (Petrone et al. 2026b). The following table describes the structure of the occurrence data. The dataset submitted to GBIF includes all records for which a taxonomic identification to species (or lower) level was possible. It is structured as an occurrence table published as a Darwin Core Archive (DwCA), which is a standardised format for sharing biodiversity data as a set of one or more data tables. The core data file contains 11023 records for Pico Island (occurrenceID). This GBIF IPT (Integrated Publishing Toolkit, Version 2.5.6) archives the data and, thus, serves as the data repository. The data and resource metadata are available for download in the Portuguese GBIF Portal IPT (Petrone et al. 2026b).

**Data set 2.** 

Column label	Column description
eventID	An identifier for the set of information associated with a dwc:Event (something that occurs at a place and time). May be a global unique identifier or an identifier specific to the dataset.
type	The nature of the resource.
licence	A legal document giving official permission to do something with the resource.
institutionID	The identifier for the institution having custody of the object or information referred to in the record.
institutionCode	The acronym of the institution having custody of the object or information referred to in the record.
occurrenceID	Identifier of the record, coded as a global unique identifier.
basisOfRecord	The specific nature of the data record.
dynamicProperties	A list of additional measurements, facts, characteristics or assertions about the record. Meant to provide a mechanism for structured content.
establishmentMeans	Statement about whether a dwc:Organism has been introduced to a given place and time through the direct or indirect activity of modern humans.
recordedBy	A list (concatenated and separated) of names of people, groups or organisations who performed the sampling in the field.
identifiedBy	A person, group or organisation who assigned the dwc:Taxon to the subject.
dateIdentified	The date on which the subject was determined as representing the dwc:Taxon.
scientificName	Complete scientific name, including author and year.
kingdom	Kingdom name.
phylum	Phylum name.
class	Class name.
order	Order name.
family	Family name.
genus	Genus name.
specificEpithet	Specific epithet.
infraspecificEpithet	Infraspecific epithet.
taxonRank	Lowest taxonomic rank of the record.
scientificNameAuthorship	Name of the author of the lowest taxon rank included in the record.

## Additional information

**Figure 3.**
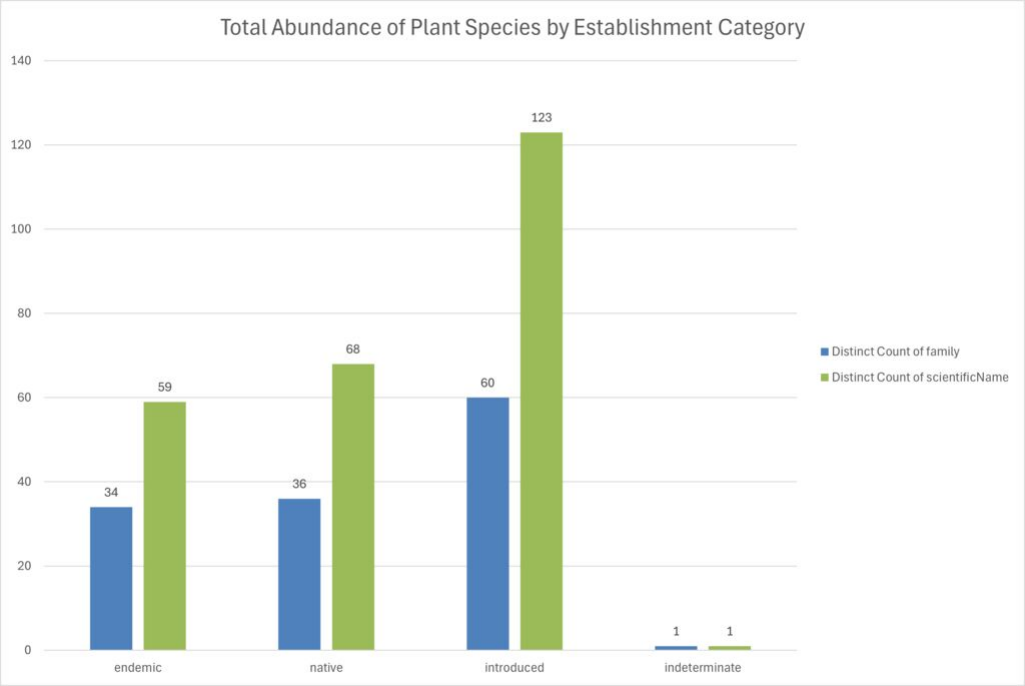
Number of distinct vascular plant species (scientificName) and families (family) recorded on Pico Island, categorised by floristic status (endemic, native, introduced and indeterminate).

**Figure 4.**
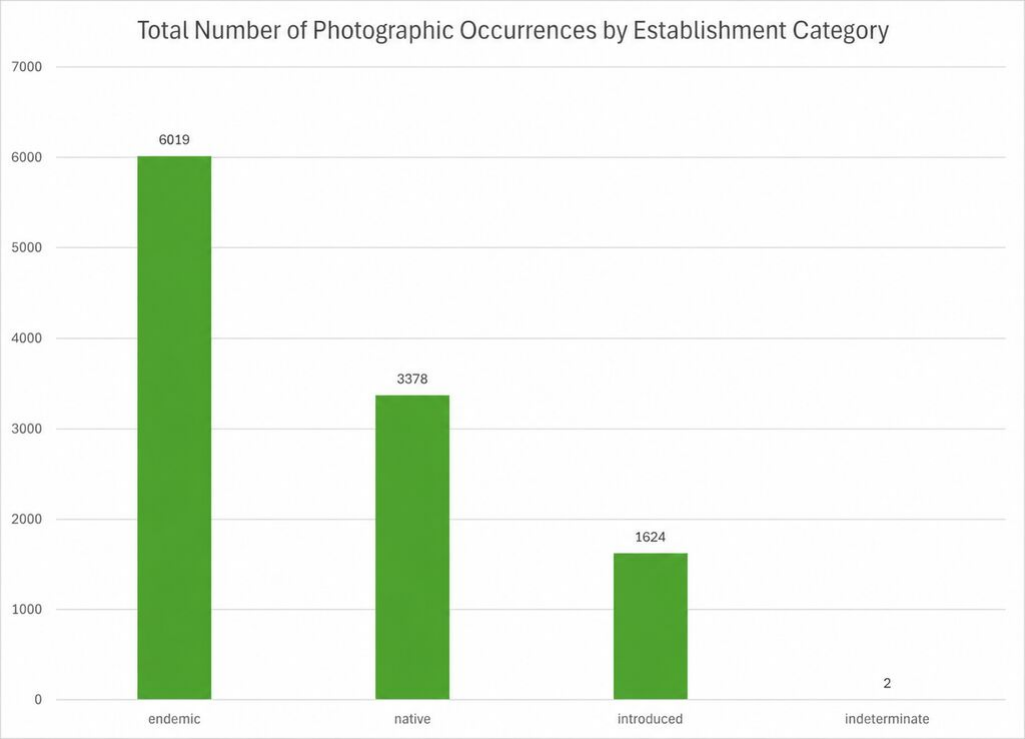
Total number of photographic occurrences of vascular plant species per floristic status, reflecting relative abundance across surveyed locations in Pico Island.

A comprehensive survey of Pico Island’s vascular plant flora yielded 251 distinct taxa, representing six classes, 38 orders and 88 families (Suppl. material 1). An overview of the distribution of establishment categories is provided in Figs 3, 4.

**Figure 5.**
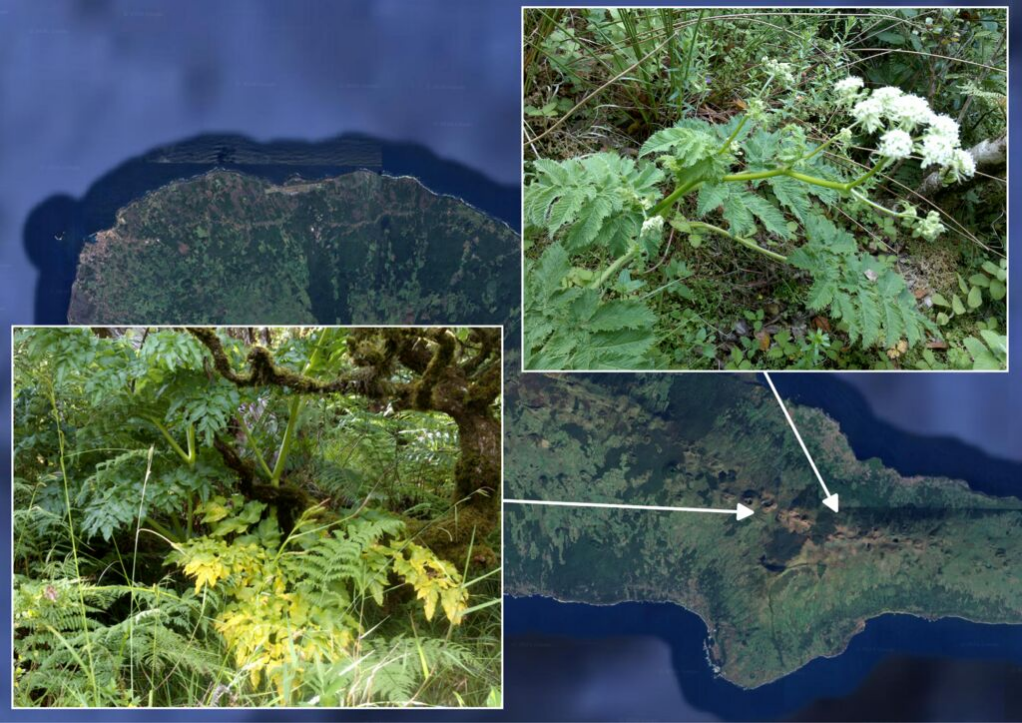
*Angelica lignescens* Reduron & Danton (left) and *Chaerophyllum azoricum* Trel. (right), two Azorean endemics of humid montane habitats. *Angelica lignescens* grows mainly in woodlands, bogs and wet slopes (500–900 m a.s.l.), whereas *Chaerophyllum azoricum* occurs in grasslands, scrublands, ravines, craters and cliffs (600–1000 m a.s.l.).

**Figure 6.**
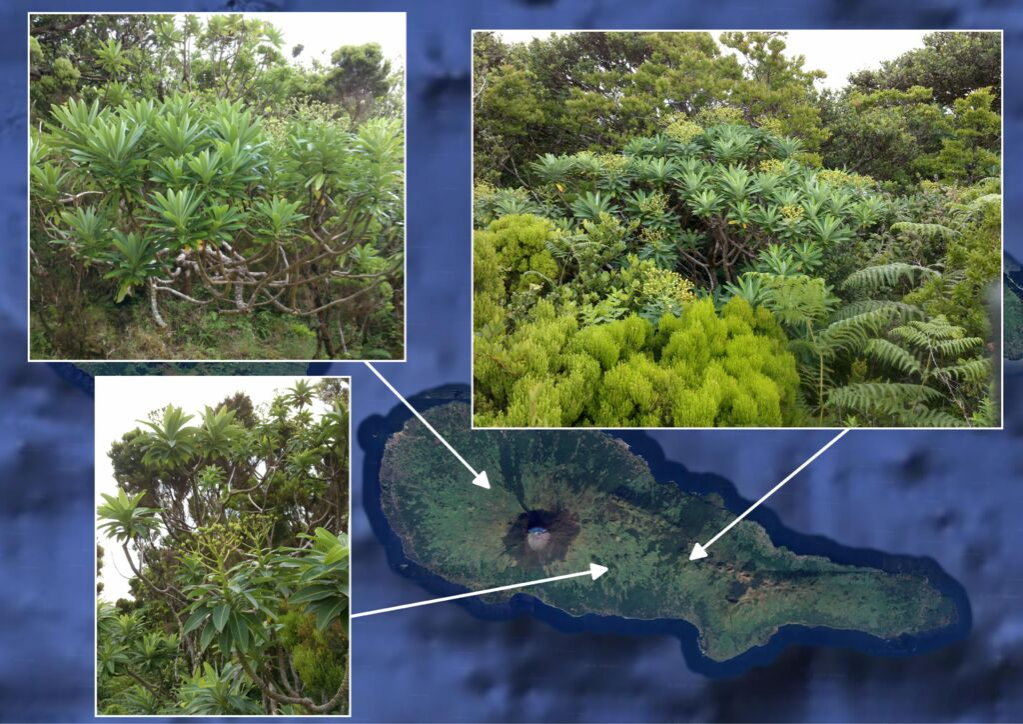
*Euphorbia stygiana* H.C.Watson subsp. stygiana, an Azorean endemic shrub occurring in montane forests, pioneer scrublands, cliffs and volcanic craters, mainly between 600 and 900 m a.s.l. and particularly frequent on Pico Island.

**Figure 7.**
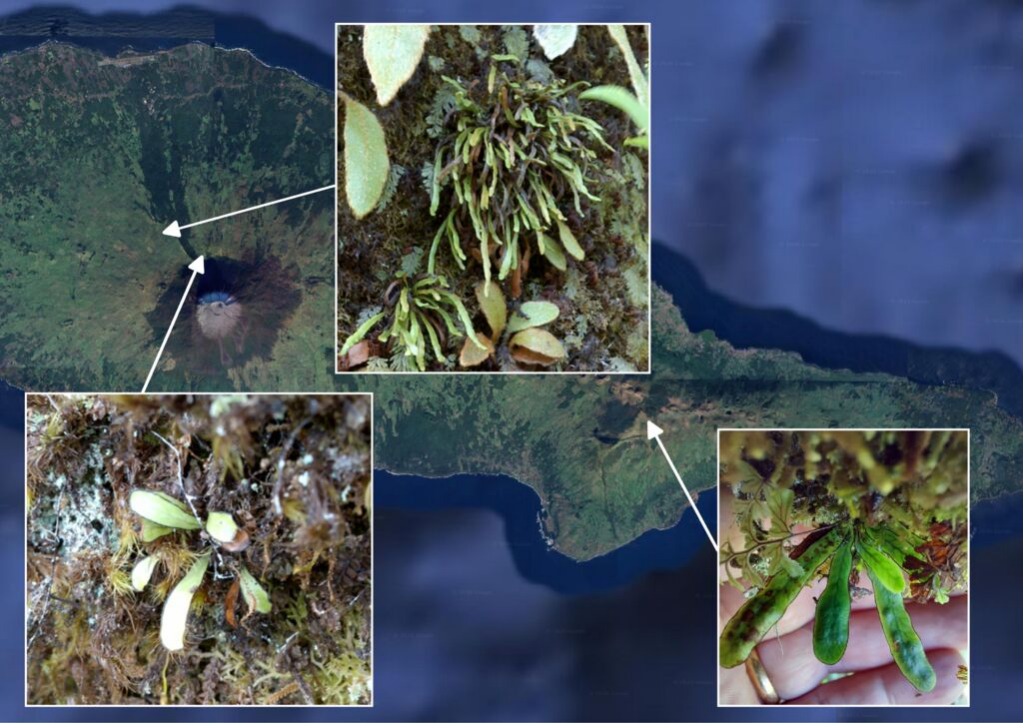
*Grammitis azorica* (H.Schaef.) H.Schaef. (right and left bottom) and *Ceradenia jungermannioides* (Klotzsch) L.E.Bishop (top centre), two rare epiphytic ferns of Pico Island's montane forests. *Grammitis azorica* is an endangered species restricted to a few known individuals (600–1000 m a.s.l.), while *Ceradenia jungermannioides* occurs in similar habitats between 600 and 900 m a.s.l.

**Figure 8.**
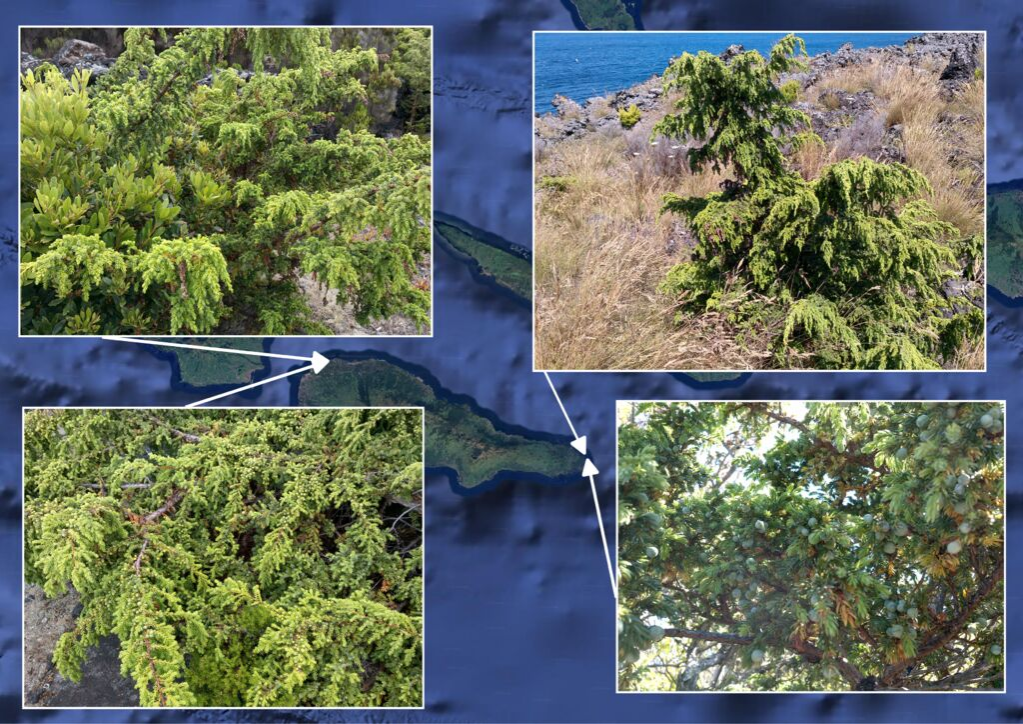
*Juniperus brevifolia* (Seub.) Antoine subsp.maritima R.B.Elias & E.Días, a coastal Azorean endemic restricted to maritime scrub communities below 100 m a.s.l., with small and isolated populations of high conservation concern.

**Figure 9.**
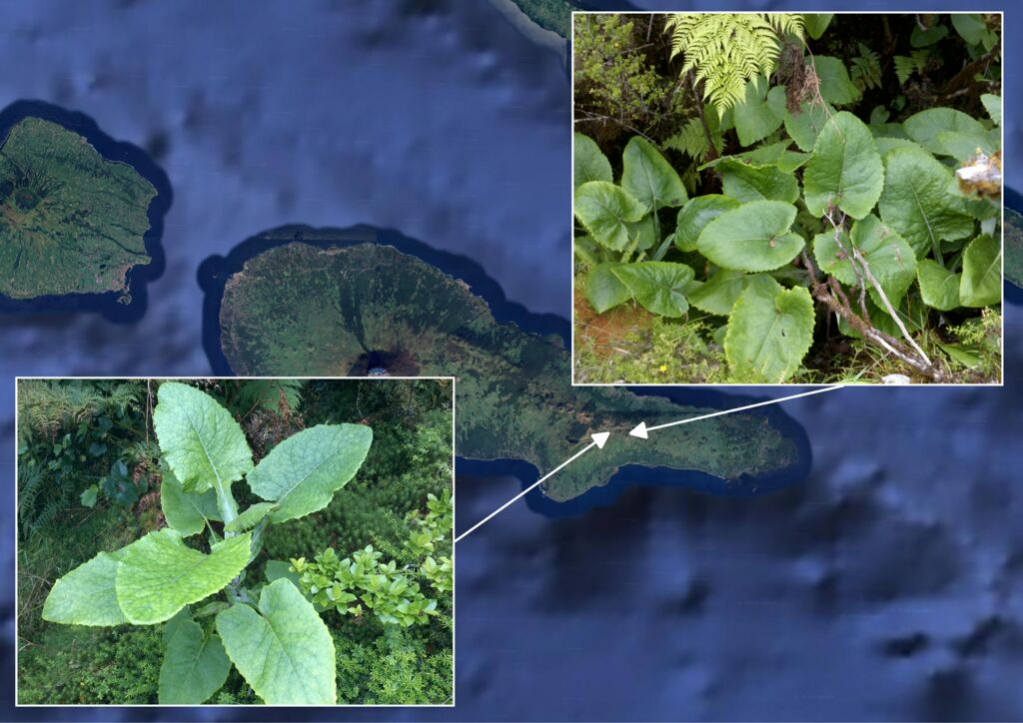
*Lactuca watsoniana* Trel., an Azorean endemic of montane forests, volcanic craters and ravines, mainly between 600 and 1000 m a.s.l. and particularly frequent on Pico Island and Terceira.

**Figure 10.**
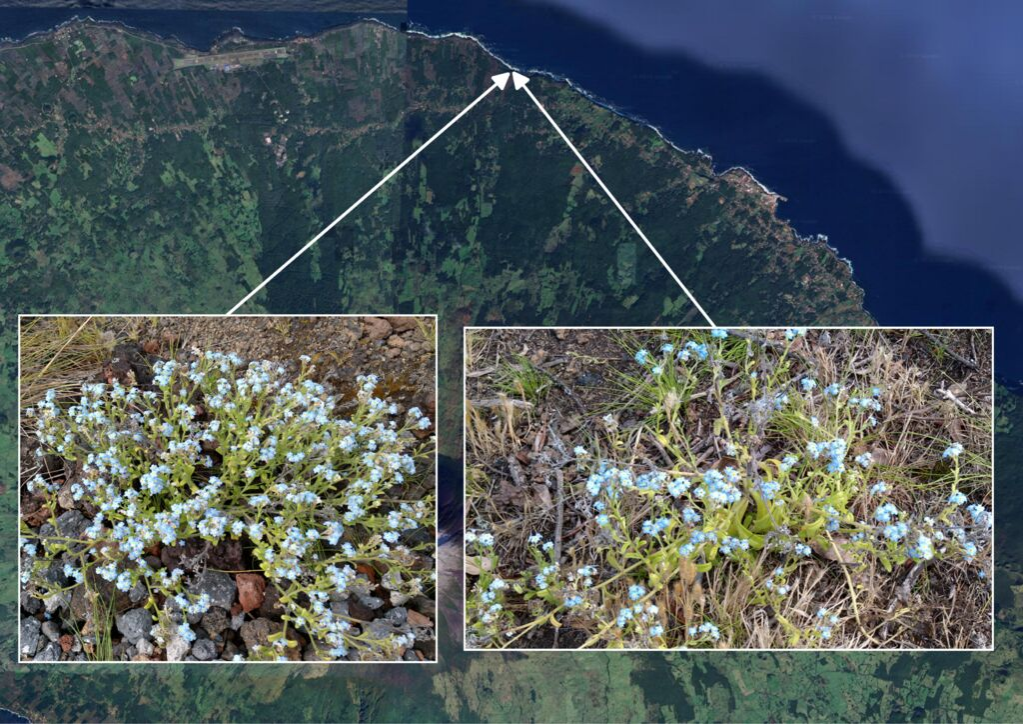
*Myosotis maritima* Hochst. ex Seub., a coastal Azorean endemic of supralittoral zones, rocky cliffs and exposed slopes below 50 m a.s.l., with ascending, highly branched stems.

**Figure 11.**
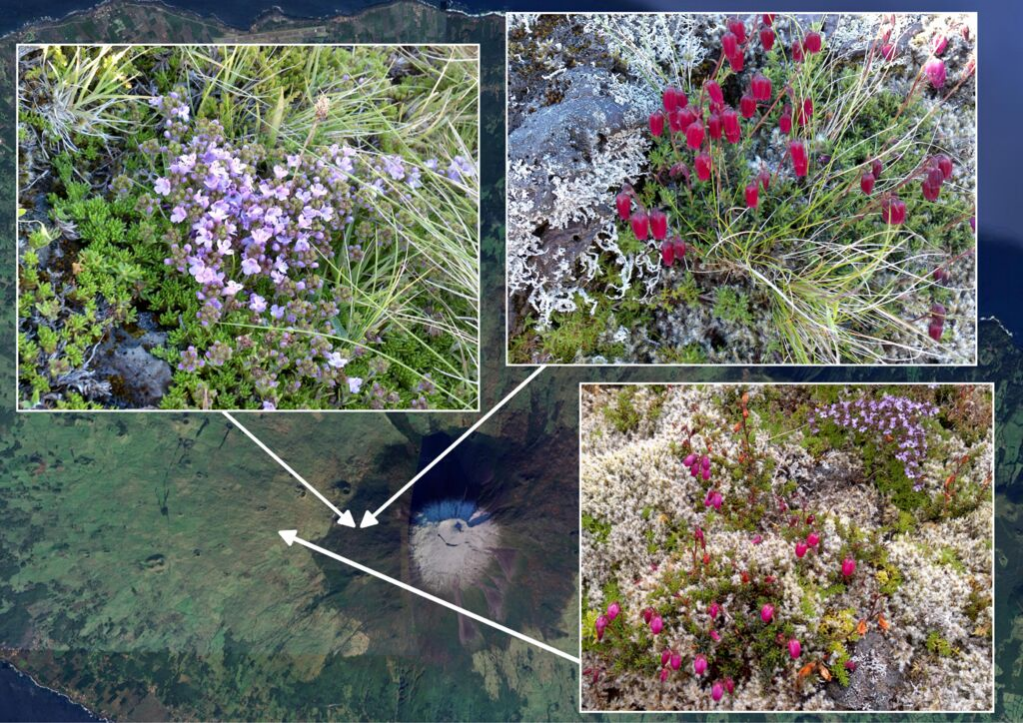
*Daboecia azorica* Tutin & E.F.Warb., a small evergreen shrub of subalpine and alpine scrublands (600–2200 m a.s.l.) and *Thymus caespititius* Brot., a native prostrate shrub of rocky habitats occurring from the coast to the summit zone up to 2200 m a.s.l.

**Figure 12.**
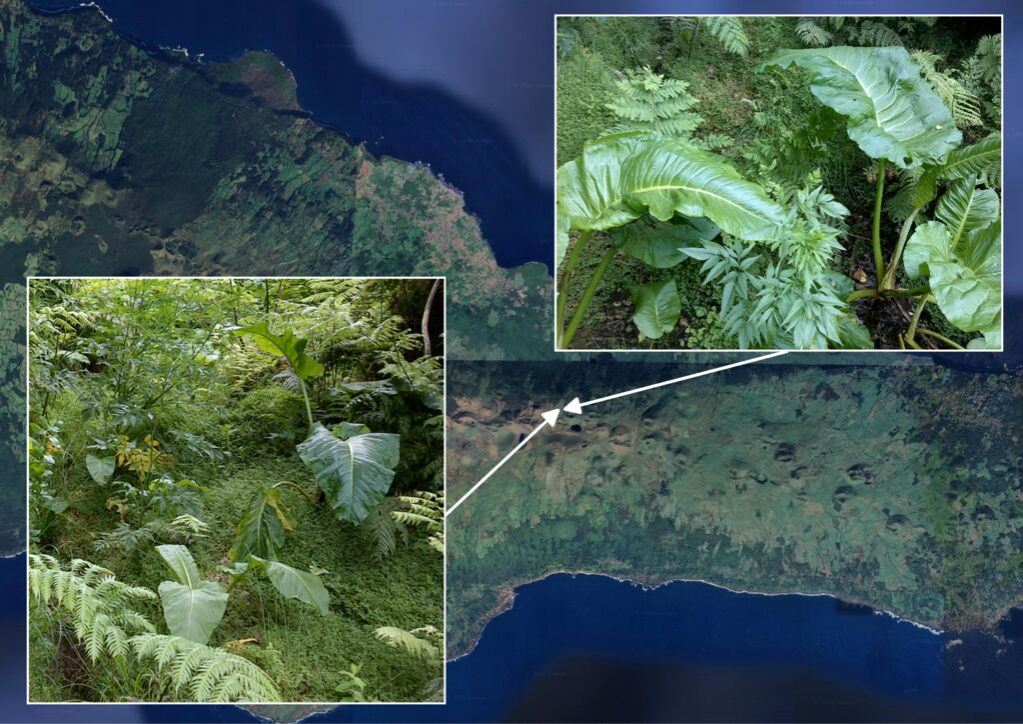
*Rumex azoricus* Rech.f. is a large endemic perennial species occurring mainly in native grasslands and along stream margins up to 950 m a.s.l., characterised by erect, branched stems.

**Figure 13.**
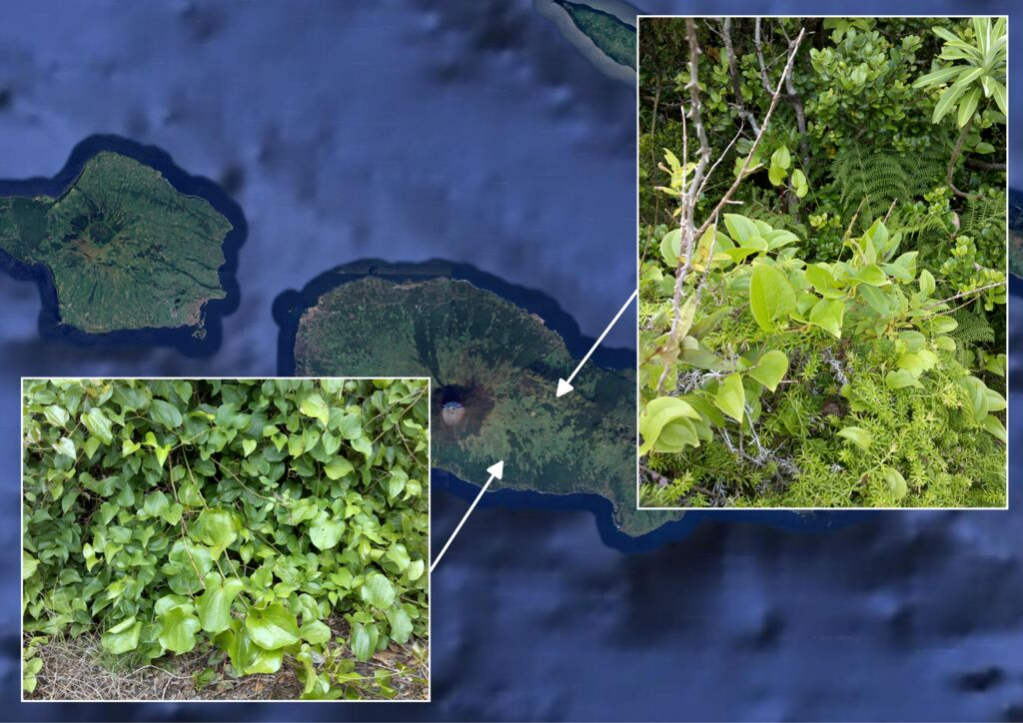
*Smilax azorica* H.Schaef. & P.Schönfelder, a dioecious woody climber endemic to the Azores, generally rare or scattered in humid montane forests between 200 and 1000 m a.s.l.

Pico Island hosts a remarkable diversity of habitats, ranging from coastal lava fields and lowland vegetation to montane cloud forests and high-elevation volcanic environments. These ecosystems support complex plant communities and several endemic and native species of high conservation value, including *Angelica lignescens* Reduron & Danton and *Chaerophyllum azoricum* Trel. (Fig. 5), *Euphorbia stygiana* H.C.Watson subsp. stygiana (Fig. 6), *Grammitis azorica* (H.Schaef.) H.Schaef. and *Ceradenia jungermannioides* (Klotzsch) L.E.Bishop (Fig. 7), Juniperus brevifolia subsp. maritima R.B.Elias & E.Días (Fig. 8), a rare coastal endemic taxon restricted to low-elevation maritime scrub communities (Elias and Dias 2014), *Lactuca watsoniana* Trel. (Fig. 9), *Myosotis maritima* Hochst. ex Seub. (Fig. 10), *Daboecia azorica* Tutin & E.F.Warb. and *Thymus caespititius* Brot. (Fig. 11), *Rumex azoricus* Rech.f. (Fig. 12) and *Smilax azorica* H.Schaef. & P.Schönfelder (Fig. 13). The information regarding species ecology and distribution were obtained from Elias et al. (2024).

## Supplementary Material

E929FF3A-D478-5ACC-A75A-43B83D7D0571 10.3897/BDJ.14.e206276.suppl1 Supplementary material 1 Table with the list of species for Pico Island Data type taxonomic list Brief description This supplementary table provides the complete list of vascular plant taxa recorded in Pico Island, including their colonisation status and, for each species or subspecies, the corresponding number of occurrences, presented in the column 'Count', giving an idea of how widespread the taxon is on the Island. File: oo_1705055.csv https://binary.pensoft.net/file/1705055 Andrea Petrone, Paulo A.V. Borges, Fernando Pereira, Rui B. Elias
